# Revealing the Hidden Details of Nanostructure in a Pharmaceutical Cream

**DOI:** 10.1038/s41598-020-61096-x

**Published:** 2020-03-05

**Authors:** Delaram Ahmadi, Najet Mahmoudi, Peixun Li, Kun Ma, James Doutch, Fabrizia Foglia, Richard K. Heenan, David Barlow, M. Jayne Lawrence

**Affiliations:** 1 Institute of Pharmaceutical Science, King’s College London, Franklin Wilkins Building, 150 Stamford Street, London, SE1 9NH UK; 2 STFC ISIS Facility, Rutherford Appleton Laboratory, Chilton, Didcot, OX11 0QX UK; 3 Department of Chemistry, Christopher Ingold Laboratories, University College London, Gordon Street, London, WC1H 0AJ UK; 4 Division of Pharmacy & Optometry, Stopford Building, University of Manchester, 99 Oxford Road, Manchester, M13 9PG UK

**Keywords:** Colloids, Gels and hydrogels, Surfaces, interfaces and thin films

## Abstract

Creams are multi-component semi-solid emulsions that find widespread utility across a wide range of pharmaceutical, cosmetic, and personal care products, and they also feature prominently in veterinary preparations and processed foodstuffs. The internal architectures of these systems, however, have to date been inferred largely through macroscopic and/or indirect experimental observations and so they are not well-characterized at the molecular level. Moreover, while their long-term stability and shelf-life, and their aesthetics and functional utility are critically dependent upon their molecular structure, there is no real understanding yet of the structural mechanisms that underlie the potential destabilizing effects of additives like drugs, anti-oxidants or preservatives, and no structure-based rationale to guide product formulation. In the research reported here we sought to address these deficiencies, making particular use of small-angle neutron scattering and exploiting the device of H/D contrast variation, with complementary studies also performed using bright-field and polarised light microscopy, small-angle and wide-angle X-ray scattering, and steady-state shear rheology measurements. Through the convolved findings from these studies we have secured a finely detailed picture of the molecular structure of creams based on Aqueous Cream BP, and our findings reveal that the structure is quite different from the generic picture of cream structure that is widely accepted and reproduced in textbooks.

## Introduction

Oil-in-water creams are widely used in pharmaceutical products formulated to treat conditions like atopic dermatitis, rosacea and psoriasis^1^. They also form the basis of veterinary medications used to assist wound-healing and treat skin infections^2^, and they are the basis too of a great many personal care products, spanning make-up foundations, anti-perspirants, sun lotions and deodorants^3^. All such products are complex, multi-component colloids in which the constituent oil and water phases are stabilised through the addition of one or more ionic, non-ionic or zwitterionic surfactants, in combination with one or more co-surfactants (sometimes referred to as consistency enhancers) which might be long chain fatty acids, fatty alcohols or monoglycerides^4,5^. Other components which might be added include humectants such as propylene glycol, anti-oxidants like α-tocopherol acetate, and preservatives like phenoxyethanol^6^.

The molecular architecture of any given cream formulation will clearly vary according to its chemical composition, and this in turn will determine its chemical and physical stability, and also impact the cream’s sensory aesthetics and efficacy as a cosmetic, pharmaceutical, or veterinary product. Despite much research into the properties of creams and their many years of use, however, our understanding of their internal *molecular* structure is still far from complete, with the unavoidable consequence, therefore, that they are still formulated only in a semi-empirical manner.

Junginger and co-workers^7^ were the first to suggest a model of cream structure, on the basis of their findings from a combination of X-ray diffraction, microscopy, and differential scanning calorimetry measurements. The model they proposed involved a gel network of surfactant/co-surfactant bilayers formed within the aqueous phase of the system, with excess added co-surfactant(s) giving rise to bilayers which serve to surround and immobilize the oil droplets that are otherwise enveloped and held dispersed by a surfactant/co-surfactant monolayer. Eccleston and Barry elaborated Junginger’s proposals and provided a coherent explanation of the mechanism by which the excess surfactant and co-surfactant not only provide stability but also modulate the cream viscosity^8^. In more recent studies reported, it has been proposed that other types of structures might exist in oil-in-water creams. Kónya *et al*.^4^ have suggested that those prepared with isopropyl myristate, glyceryl stearate and cetostearyl alcohol contain surfactant micelles dispersed within the aqueous phase, and Dahl *et al*.^9^ have proposed that those prepared from polyglyceryl-3-distearate, octadecanol and hexadecanoic acid 2-ethylhexyl ester contain multi-lamellar vesicles.

It would thus seem that different compositions of creams might have rather different internal architectures, but it is important to note here that in all studies on creams reported to date, much of the molecular structural detail has been inferred from *indirect* observations with precious little determined by direct means. Normal light microscopy is regularly used to distinguish features on the micron scale, with polarizing filters often employed to show birefringence and so to infer the presence of molecular ordering (*c.f*.,^10,11^). These techniques probe only at the macroscopic level, however, and the nature and composition of the cream’s internal interfaces and the nature of the ordered systems that gives rise to the birefringence can only be inferred and not proven. In like manner, researchers use wide-angle X-ray scattering^12^ and differential scanning calorimetry^10,13–16^ to provide details of the phase state and lateral organisation of a cream’s excipients but neither of these techniques can inform as to the nature of the aggregates formed by the excipients and they cannot be used to pinpoint their microstructural loci.

The form and make-up of a cream’s various internal interfaces, and the morphology, size, and composition of any aggregates present, can only be probed *directly* through small-angle X-ray scattering (SAXS) or small-angle neutron scattering (SANS) studies, and it is the latter that is the more generally useful because of the high contrast achieved by using protiated organic excipients formulated with D_2_O. Moreover, if the SANS studies are performed so as to exploit the device of hydrogen/deuterium contrast variation, experiments can be performed that allow selected cream components to be explicitly highlighted and the nature of their organisation and their loci within the cream’s microstructure can be demonstrated unequivocally.

In the studies reported here we have made particular use of SANS to probe the molecular architecture of an ostensibly simple pharmaceutical cream based on Aqueous Cream BP^17,18^, comprising liquid paraffin, water, sodium dodecyl sulfate surfactant, and a (1:1) mixture of the co-surfactants, cetyl and stearyl alcohol. Complementary studies have also been performed using a combination of bright-field and polarized light microscopy, SAXS/WAXS experiments, and variable shear rheology measurements.

## Results

**Figure 1:**
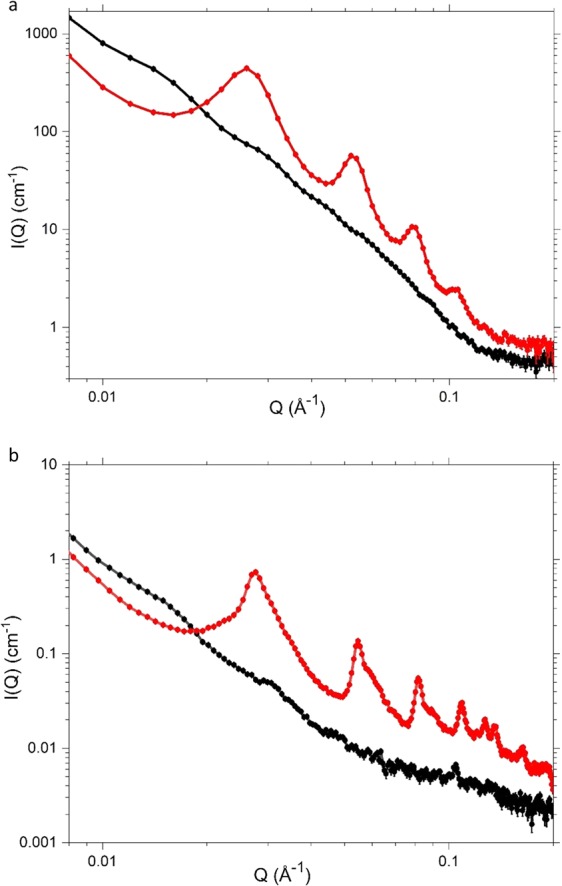
SANS (**a**) and SAXS (**b**) profiles measured at 25 °C for 4% (C1; black) and 10% (C2; red) emulsifier creams. Cream compositions are as detailed in Methods, Table 1. Standard errors on the measured data are shown but are subsumed within the plotted symbols.

The SANS and SAXS profiles of scattering intensities (I(Q) *vs* momentum transfer, Q = 4πsinθ/λ) obtained for the cream containing 10% w/w surfactant and co-surfactants show sharp and well-defined Bragg peaks with 4-5 orders of reflection, collectively fitted to a *d*-spacing (at 25 °C) of 237 ± 1 Å with a (Schultz) polydispersity of 0.04 (Fig. 1).

**Figure 2:**
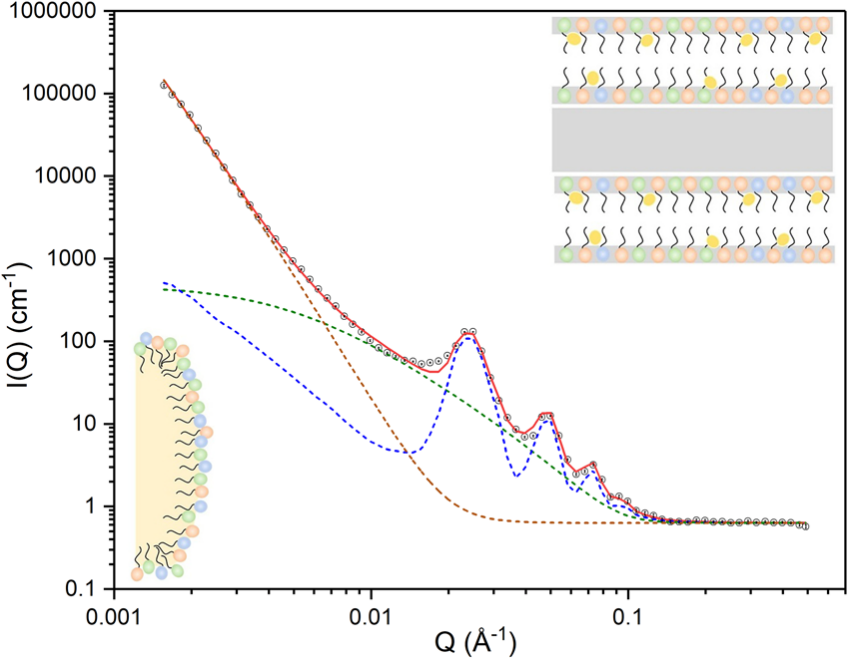
SANS profile (black circles) measured at 25 °C for the 10% w/w emulsifier cream with the model-fitted curve (red) calculated assuming a para-crystalline lamellar stack with a mean of twenty, 49 Å thick bilayers *per* stack and a *d*-spacing of 237 Å (dashed blue line) combined with some individual/unstacked bilayers with the same dimensions (dashed green line) and a power law modelling of the scattering (dashed brown line), with the fitted value of the power law exponent (−4.8 ± 0.1) taken to indicate a diffuse interfacial surfactant/co-surfactant layer at the surface of the oil droplets. Insets give schematic illustrations (not to scale) of a pair of co-surfactant bilayers (top right) and the surfactant/co-surfactant layer covering an oil droplet (bottom left).

Analytical modelling of the whole SANS profile (Fig. 2) is achieved to a high goodness-of-fit assuming a para-crystalline lamellar stack (consistent with the Maltese crosses evident in the polarised light micrographs; Fig. S2, Supplementary information) with a mean of twenty stacked layers, each of thickness, 49 ± 1 Å, combined with some individual/unstacked layers with the same dimensions, and a power law modelling to account for the steep rise in *I*(*Q*) below *Q* = 0.01 Å^−1^ ^19–21^. The power law exponent is fitted as −4.8 ± 0.1, and this testifies to a diffuse boundary between two phases^19,22^, which we here take to describe the interfacial surfactant/co-surfactant layer that coats the micron-sized oil droplets dispersed within the cream (and gives rise to the birefringence seen in the polarised light micrographs; Fig. S2, Supplementary information). Modelling of the corresponding SAXS data (not shown) confirms the SANS estimate for the lamellar *d*-spacing (237 ± 1 Å, with a polydispersity of 0.04) and also supports the presence of a diffusive interface (with a fitted power law exponent of −4.5 ± 0.1). The agreement so obtained in fitting the SANS and SAXS data gives confidence in the model used, and also testifies to the absence of H/D isotope effects which might otherwise confound interpretation of the SANS contrast match experiments (*vide infra*).

A similar lamellar ordering is seen in the corresponding oil-free (ternary) system but here the SANS profile is modelled without the need to include a power law dependence of the low-*Q* scattering, and with the lamellar stacks having an average of five layers, each with a thickness of 46 ± 1 Å, and with a larger *d*-spacing of 265 ± 1 Å with a polydispersity of 0.09 (see Fig. S3, Supplementary information).

The 46 Å and 49 Å thick layers are taken to be bilayers wholly or predominantly composed of the cetyl and stearyl alcohols. These estimated layer thicknesses are consistent with the extended alkyl chain lengths for the two alcohols (21.7 Å and 24.2 Å, respectively) and they are consistent too with the bilayer thicknesses reported by Valoppi *et al*. for 5% mixtures of cetyl and stearyl alcohol blended with peanut oil (43.6 Å and 48.5 Å, respectively)^23^. The 3 Å increase in the thickness of the bilayers that we see when the ternary gel is converted to a cream suggests that the two systems have bilayers with differing compositions, and this we attribute to the incorporation of small quantities of liquid paraffin into the bilayers in the cream. Such an intrusion of oil into the lamellar bilayers has previously been suggested by Savic *et al*. on the basis of inferences drawn from their thermal analyses of creams^13^.

The fact that we see a 30 Å decrease in the *d*-spacing when oil is added to convert the ternary gel to a cream (see Fig. S5, Supplementary information) suggests that the introduction of oil causes a reduced electrostatic repulsion between the bilayers in the lamellar stacks, in part arising perhaps because of a change in surface charge density resulting from a change in molecular packing caused by the intrusion of oil into the bilayers, and otherwise accounted for by the insertion of some low dielectric material (perhaps surfactant/co-surfactant bicelles, *vide infra*) within the otherwise high dielectric inter-lamellar water layers.

When the cream is prepared using only 4% w/w surfactant and co-surfactants, the SANS and SAXS profiles show only the vestiges of Bragg reflections (Fig. 1), testifying to a much-reduced volume fraction of the para-crystalline lamellar network by comparison with that seen in the 10% w/w cream. Consistent with the proposal put forward by Eccleston *et al*.– that the extent of the lamellar network determines the “body” of a cream^24^ – we thus find that the SANS profile for the 4% w/w cream and its corresponding ternary system can be model-fitted (see Fig. S6, Supplementary information) assuming lamellar stacks with a mean of just two bilayers per stack (but with the same thicknesses as found for the 10% w/w systems), and rheology measurements show that the 4% w/w cream exhibits a much lower viscosity than the 10% w/w cream, the viscosity indices for the two creams estimated as 61 Pa.s^−1^ and 121 Pa.s^−1^, respectively (see Fig. S4, Supplementary information).

**Figure 3:**
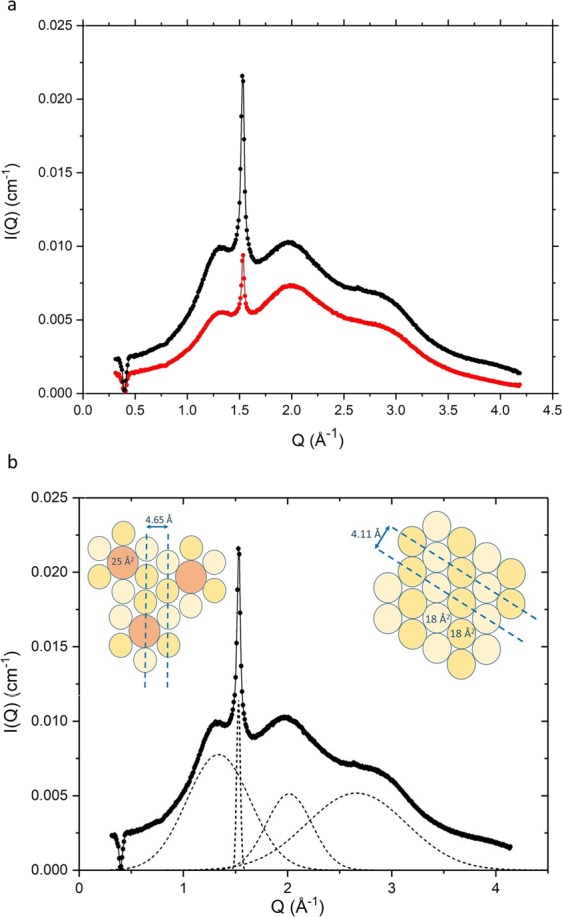
WAXS profiles measured at 25 °C for 4% (C1) (red) and 10% (C2) (black) emulsifier creams (**a**) and the deconvolution of the profile for the 10% cream, revealing peaks corresponding to lateral spacings (left to right) of 4.65 Å, 4.11 Å, 3.12 Å and 2.36 Å (**b**). Insets show the hexagonal packing of the cetyl and stearyl alcohols (interfacial areas, 18 Å^2^) in the bilayers of the lamellar stack, and the distorted hexagonal packing of the alcohols and SDS (interfacial area, 25 Å^2^ ^42^) in the layer surrounding each oil droplet. Cream compositions are as detailed in Methods, Table 1.

The WAXS patterns obtained for the 4% w/w and 10% w/w emulsifier creams (*cf*. Fig. 3) both give rise to a sharp reflection at 4.11 Å which is indicative of a liquid crystalline (*L*_α_) gel phase, and testifies to alkyl chains that are packed in an hexagonal array and have freedom to rotate about their long axes^12,13,25–28^. The same sharp reflection is seen in the WAXS patterns recorded for the 10% w/w ternary system (see Fig. S7, Supplementary information). In the creams, we attribute the 4.11 Å reflection to the lamellar bilayers which we consider contain cetyl and stearyl alcohol with negligible SDS (*vide infra*) and in the ternary systems we suggest that it arises from bilayers of the two co-surfactants mixed with SDS. Deconvolution of the WAXS profiles for the creams also reveals that there are other much broader peaks observed with spacings of 2.36 Å, 3.12 Å, and 4.65 Å. The shorter two of these broad peak spacings are taken to arise as a function of the interatomic distributions of O-O, O-H and H-H for the water in the formulations, since they exactly match the spacings for liquid water that have previously been reported and modelled using *ab initio* molecular dynamics simulations^26,29^. The 4.65 Å spacing – being absent from the WAXS profiles for the corresponding ternary systems (*cf*., Fig. S7, Supplementary information) – is attributed to a co-existing liquid crystalline phase in the creams, and this we take to arise from a loosely packed mixed layer of the SDS and cetyl and stearyl alcohols surrounding the oil droplets.

**Figure 4:**
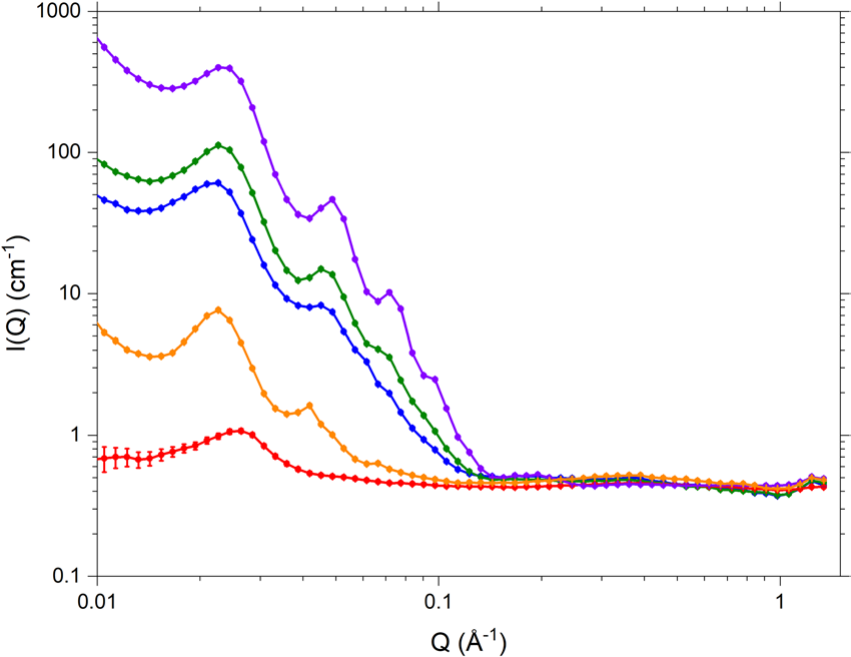
SANS profiles measured at 25 °C for 10% emulsifier creams prepared with all components protiated in D_2_O (C12) purple) and with selected individual excipients deuterated (*d*_33_-hexadecanol (C6), blue; *d*_37_-octadecanol (C7), green; *d*_25_-SDS (C9), red; *d*_10_-1,5-pentanediol (C11), orange) and all other (protiated) components contrast-matched with a solvent of 96% H_2_O/4% D_2_O. Cream compositions are as detailed in Methods, Table 2.

In order to determine the microstructural loci of the individual cream constituents, further SANS measurements were made on formulations prepared with one of the constituents perdeuterated (either *d*_25_-SDS, *d*_33_-cetyl alcohol, or d_37_-stearyl alcohol) and all the remaining (protiated) organic excipients made effectively invisible to neutrons through contrast-matching with an aqueous phase comprising 96% H_2_O and 4% D_2_O. The resulting SANS profiles for the formulations containing perdeuterated cetyl and stearyl alcohols both show well defined Bragg peaks (Fig. 4), and the model-fitted bilayer thicknesses and *d*-spacings match from one profile to the other and also match those determined in model-fitting the data for the cream with all organic constituents fully protiated (data not shown). By this means, therefore, we provide the first *direct* evidence for the co-location of the two co-surfactants within the bilayers of the para-crystalline stacks, and from the volume fractions estimated through the model-fitting, we find that the two alcohols are more-or-less equally represented in the bilayers.

**Figure 5:**
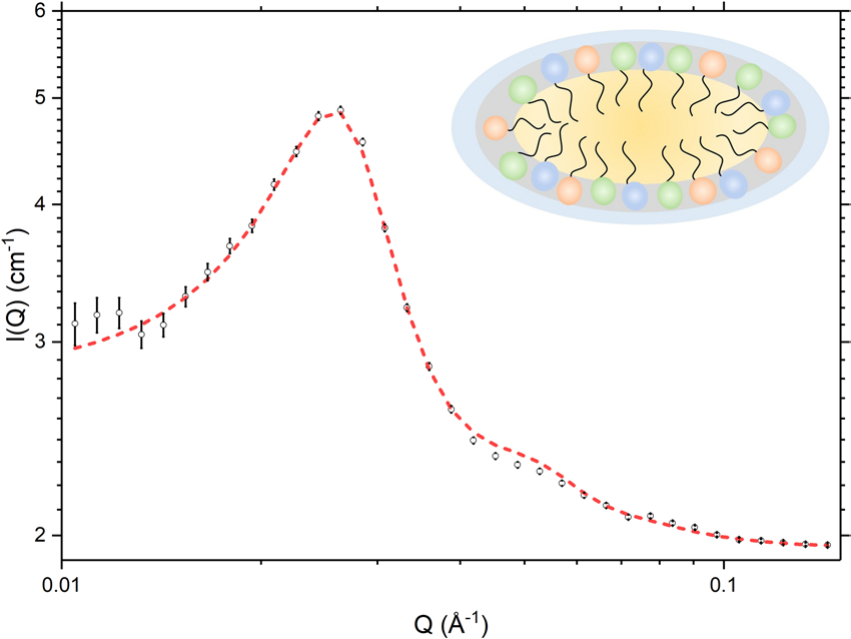
SANS profile recorded at 25 °C for a 10% emulsifier cream containing 1% *d*_25_-SDS and with the oil and co-surfactants protiated and contrast-matched with an aqueous phase comprising 96:4 v/v H_2_O:D_2_O (C9). The experimental data (black) are model-fitted (red) assuming (negatively) charged oblate ellipsoidal aggregates with dimensions, 120 Å × 120 Å × 22 Å. Inset shows a schematic of the bicelle (not to scale). Cream composition as detailed in Methods, Table 2.

The corresponding contrast-matched cream prepared with (1% w/w) *d*_25_-SDS, however, gives a quite different SANS profile (Fig. 5) that cannot be modelled assuming a para-crystalline stack of bilayers, nor by assuming a polydisperse system of spherical particles. These data are instead model-fitted assuming oblate ellipsoidal aggregates, with a 4 Å thick shell and a central core having equatorial radii of 120 Å and a polar radius of 22 Å. The aggregates carry a high surface charge (of the order of 200 per aggregate) that gives rise to strong inter-particulate interactions which we model with a Hayter-Penfold structure factor^30,31^. Given the shape and size of these aggregates, it is clear that they cannot be SDS micelles, which (at this concentration) are generally reported as prolate ellipsoids with dimensions of the order of 17 Å × 17 Å × 25 Å^32,33^. On the basis of the model-fitted semi-axes dimensions and the scattering length densities of their core and shell (1.91 × 10^−6^ Å^−2^ and 3.59 × 10^−6^ Å^−2^, respectively), we propose in fact that these aggregates are corpuscular/disc-shaped bicelles comprising only 30% SDS by volume, with the remaining 70% made up of a 1:1 mixture of cetyl and stearyl alcohol. The weighted mean interfacial surface area (*a*_0_), chain length (*l*) and hydrophobe volume (*v*) for such a mixture gives the critical packing parameter (*v*/*a*_0_*l*) as 0.78, and this is as would be predicted for non-planar, flexible bilayers^34^.

**Figure 6:**
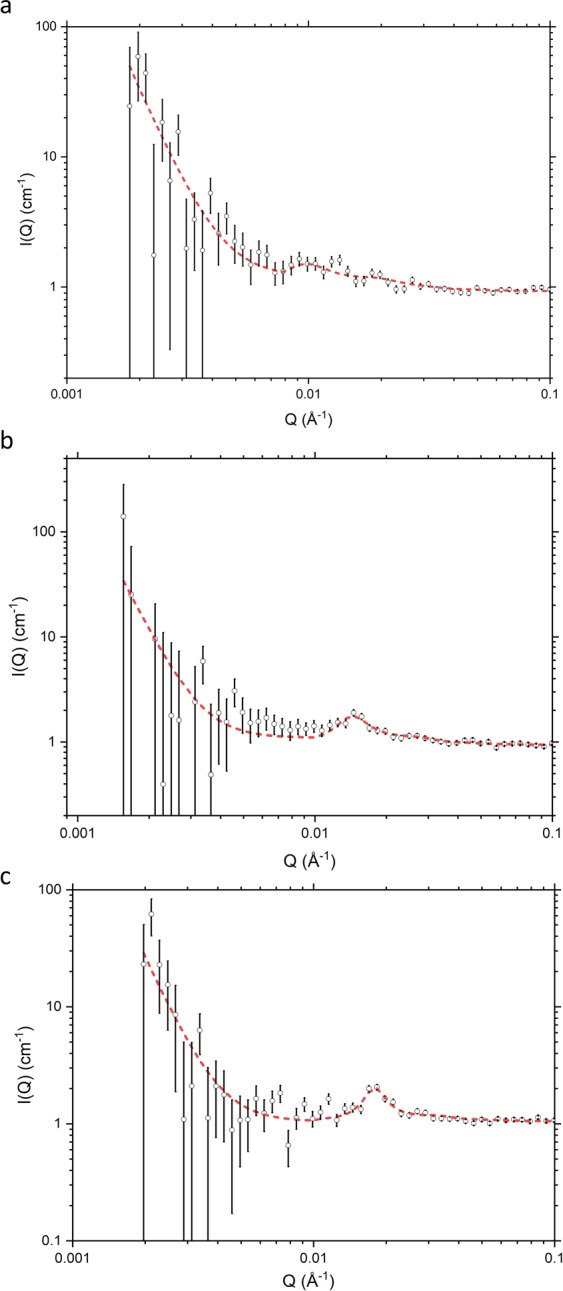
SANS profiles recorded at 25 °C for creams prepared with 4% (C3), (**a**); 5% (C4), (**b**); and 7% (C5), (**c**) emulsifier and containing 1% SDS (half of which was *d*_25_-labelled) in solvent contrast-matched to all other organic excipients with an aqueous phase comprising 97:3 v/v H_2_O:D_2_O. The experimental data (black) are model-fitted (red) assuming (negatively) charged oblate ellipsoidal aggregates. Cream compositions are as detailed in Methods, Table 2.

When the SDS:alkanols ratio is maintained as 1:9 and the total emulsifier content of the contrast-matched creams containing *d*_25_-SDS is reduced from 10% w/w to 7% w/w, through 5% w/w, and down to 4% w/w, the SANS profiles obtained (Fig. 6) show evidence of the same type of mixed micellar aggregate, and because these profiles were measured to lower *Q*, there is an indication of an upturn revealed in the scattering below *Q* = 0.01 Å^−1^, which we model using a power law. Whereas the contrast-matched 10% w/w emulsifier cream was prepared with all (of the 1% w/w) SDS as *d*_25_-SDS – and thus gave a SANS profile with good data statistics – the profiles obtained for the contrast-matched creams with lower emulsifier contents were prepared with only half of their (1% w/w) SDS content as *d*_25_-SDS. In consequence of this difference, the contrast-matched creams with low levels of emulsifier have a much reduced neutron scattering contrast and this conspires with their reduced total emulsifier content (and the change in sample-to-detector distance from 8 m to 12 m) to give poorer data statistics. While we were thus able confidently to model the SANS profile for the 10% w/w contrast-matched cream (containing 1% w/w SDS) allowing free optimisation of the various model parameters and using a core-shell ellipsoidal model, we considered it prudent to model the data for the corresponding creams with the lower emulsifier contents in a more parsimonious manner, using a simpler, uniform ellipsoid model, and with the ellipsoid dimensions constrained in such a way that the body of data tells a coherent and physically plausible story. In our modelling of the data for the contrast-matched 4% w/w, 5% w/w, and 7% w/w emulsifier creams we thus assume charged oblate ellipsoids, with dimensions, 120 × 120 × 22 Å, with the rise in scattering below *Q* = 0.01 Å^−1^ modelled with a power law, taking the value of the exponent as 4.5, to be consistent with the presence of a diffuse boundary between the oil and water, as seen in the data for the creams prepared with protiated organic excipients dispersed in D_2_O. The fits so obtained (Fig. 6) yield model fitted scale factors that show that the volume fractions of the bicelles decrease as the level of emulsifier is reduced.

When the contrast-matched 10% w/w emulsifier creams containing *d*_25_-SDS are prepared with 0.5% w/w or 2% w/w rather than 1% w/w SDS – such that the SDS:alkanols w/w ratio varies as 1:19 and 1:4 (*vs*., 1:9 for the cream containing 1% w/w SDS) – the resulting SANS profiles can again be modelled assuming a power law fit to the data below *Q* = 0.01 Å^−1^, and the peak in the scattering profile is fitted assuming charged oblate ellipsoidal bicelles (see Fig. S8, Supplementary information). As previously, the power law exponent is fitted with a value of *ca*. 4.5, and the bicelle dimensions are 120 × 120 × 22 Å. The fitted volume fractions of the bicelles are seen to decrease as the level of SDS is decreased.

When the humectant^35^ and anti-microbial preservative^36^ 1,5-pentanediol, is added to the 10% w/w emulsifier cream with 1% w/w SDS, the model-fit to the SANS profile obtained for the formulation prepared with all organic excipients protiated and dispersed in D_2_O shows quite unexpected changes including a 70 Å increase in the lamellar *d*-spacing, a 5 Å decrease in the modelled lamellar bilayer thickness, and a reduction in the exponent of the power law function used to model the low-Q data (see Fig. S9, Supplementary information). Contrary to expectations (given the polar nature of the pentanediol and its use as an antimicrobial preservative), it is also seen that the SANS profile for the contrast-matched cream containing *d*_10_-1,5-pentanediol shows the same pattern of Bragg peaks as seen for the corresponding creams prepared with the perdeuterated forms of the two long-chain alcohols (Fig. 4). It is thus concluded that the pentanediol is not confined within the aqueous phase but is instead associated with the lamellar bilayers. On the basis of the model-fitted parameters, and allowing for partitioning of the diol between the aqueous phase and the lamellae, we estimate that there is only 14% of the diol in the water and 86% associated with the lamellae. This significant association of the diol with the lamellae will undoubtedly lead to changes in the co-surfactant packing within the bilayers and this in turn will lead to the observed change in bilayer thickness.

## Discussion and Conclusions

The picture of aqueous creams that is generally accepted and widely reproduced in textbooks shows oil droplets within the emulsion stabilised by a surrounding monolayer of surfactant and co-surfactant, and with the excess added surfactant and co-surfactant forming stacks of widely-separated bilayers distributed throughout the aqueous continuous phase (*c.f*.,^37,38^).

The existence of the stacked bilayers in cream systems has previously been shown through SAXS studies (*c.f*.,^26,28^), but the composition of the lamellae has been inferred from thermal analyses and SAXS measurements made using the oil-free versions of the cream formulations^4,9,10,13,25^. The SANS profiles presented here provide very clear evidence of lamellae within the cream systems, and they also provide clear and *direct* evidence of their composition. The bilayers in the cream and those in the corresponding ternary gel are shown to be different, those within the cream being thicker than those in the ternary system, with the increased thickness taken to be caused by the incorporation of a small amount of oil within the bilayers. While the ternary systems have thus served and can continue to serve as useful models of the continuous phase in creams, it is important to recognise that they are imperfect models.

As we show here too, the lamellae within a cream may not – as they are often portrayed – contain significant amounts of both surfactants and co-surfactants. From our SANS studies performed using formulations based on Aqueous Cream BP and prepared with a single perdeuterated excipient and all other constituents contrast-matched, we provide unequivocal evidence that the bilayers within the lamellar stacks contain the two co-surfactants, cetyl and stearyl alcohol, but do not appear to contain significant amounts of the surfactant, SDS. The SANS data for the *d*_25_-SDS system is modelled instead to show the presence of corpuscular/oblate ellipsoidal aggregates which we take to be mixed SDS/co-surfactant bicelles, with around 30% SDS and 70% cetyl/stearyl alcohol. There have been no other reports of aggregates of this type being seen in aqueous creams but it is pertinent here to note that Zarbahksh *et al*. have repeatedly observed surfactant-rich layers which they speculate to contain surfactant micelles in the aqueous phase close to the surfactant monolayers formed at the decane- and hexadecane-water interfaces^22,39,40^, and Méndez-Bermúdez and Dominguez have shown through coarse grain molecular dynamics simulations that bilayer aggregates of this type are formed in aqueous dispersions of SDS and hexadecanol when the proportion of hexadecanol is high^41^. Experimentally, when the component oil and aqueous phases are mixed in the preparation of a cream, such aggregates would likely serve as intermediaries in the transfer of amphiphiles between the lamellae and the surfaces of the oil droplets. Even when the emulsifier content of the cream is reduced from 10% w/w down as far as 4% w/w, the SANS data clearly show that the ellipsoidal bicelles are still present, albeit in reduced numbers. Likewise, if the proportions of the surfactant and co-surfactants are varied, the bicelles are still in evidence, with a change in their abundance that mirrors the change in the proportion of SDS.

While the textbooks and review articles also show the surfactants and co-surfactants forming a monolayer at the surfaces of the oil droplets in a cream, we show here that this interfacial layer is very diffuse, suggesting that it is significantly roughened, with the constituent molecules not aligned but staggered. Again, this is entirely consistent with the observations made by Zarbakhsh *et al*.^22,39,40,42,43^ wherein the surfactant and lipid layers that form at oil-water interfaces are shown to exhibit a pronounced roughening which cannot be explained simply by thermal broadening and is considered to arise instead because of a staggering of the molecules’ hydrocarbon chains brought about by their dissolution within the oil and the resultant disruption of their side-by-side packing.

The consequences of incorporating additives into a cream are generally studied indirectly – by monitoring the changes that manifest in the cream’s thermal stability and rheological properties (*c.f*.,^44,45^) – and not by studying the additive’s effects on the cream’s molecular structure. In the studies reported here, however, we have shown how the addition of 1,5-pentanediol – a water-soluble material that would not be considered likely to perturb the cream structure – in fact causes very significant changes in the cream’s structure. *A priori* one would predict that the diol would be innocuously distributed within the aqueous phase of the cream, wherein its anti-microbial activity^46^ would serve to protect the cream against contaminating bacteria and fungi^36^. Our SANS studies of the contrast-matched cream formulated with *d*_10_-1,5-pentanediol, however, clearly show that the molecule associates with the bilayer lamellae, concomitantly leading to changes not only in the bilayer thickness and inter-lamellar spacing but also to the diffuse surfactant/co-surfactant layer surrounding the oil droplets. This seemingly innocuous additive, therefore, might – against all expectations – compromise cream stability.

We conclude, therefore, that what would appear to be a perfectly simple aqueous cream, with just six different chemical constituents, exhibits a far greater complexity of construction than has hitherto been recognised, with some structural features that are distinctly at odds with the picture currently accepted.

## Methods

The protiated forms of 1-octadecanol (Reagentplus, 99% purity), 1-hexadecanol (Reagentplus, 99% purity) and sodium *n*-dodecyl sulfate (SDS) (GC ≥ 98.0% purity) were purchased from Sigma-Aldrich UK Ltd. The perdeuterated forms of these compounds were provided through the ISIS Deuteration Facility (Rutherford Appleton Laboratory, Didcot, UK). Liquid paraffin was purchased from Merck & Co., Inc. Creams were prepared using ultrapure water (18.2 MΩ.cm obtained from Elga LabWater), D_2_O (99.9 atom % D, purchased from Sigma-Aldrich UK Ltd.), or a 96:4 or 97:3 v/v mixture of H_2_O:D_2_O (which gave a solvent with a neutron scattering length density to match the average scattering length density of the protiated components in those creams made with a single perdeuterated component).

Two different versions of Aqueous Cream B.P.^17,18^ (having all components protiated) were prepared as shown in Table 1. The total concentration of emulsifier (comprising hexadecanol, octadecanol and SDS) was varied as either 4% w/w or 10% w/w (formulations C1 and C2, respectively). In addition, oil-free ternary systems containing 4% and 10% w/w emulsifier were also prepared for SANS experiments – these systems containing only the surfactant and co-surfactant mixture and D_2_O as continuous phase.

**Table 1:** Chemical composition of aqueous creams prepared by Method A; see text.

	Total Emulsifier	Hexadecanol	Octadecanol	SDS	Liquid paraffin	1,5-pentanediol	H_2_O
	**Percentage % w/w**						
C1	4	1.70	1.90	0.40	20.00	—	76.00
C2	10	4.25	4.75	1.00	20.00	—	70.00

For the creams prepared with 10% total emulsifier and a single perdeuterated component (*d*_33_-hexadecanol, *d*_37_-octadecanol, *d*_25_-SDS or *d*_10_*-*1,5-pentanediol), the compositions were as given in Table 2.

**Table 2:** Chemical composition of 10% w/w emulsifier creams prepared with either deuterated *d*_33_-hexadecanol (C_16_OH), *d*_37_-octadecanol (C_18_OH), *d*_25-_SDS or *d*_10_*-*1,5-pentanediol (1,5-C_5_OH_2_) in contrast matched water, or all protiated components in 100% D_2_O. The deuterated chemicals in these formulations contain either 100% deuterated chemicals (C6, C7, C9 and C11) or a 50% mix of protiated and deuterated chemicals (C3, C4, C5, C8 and C10). Formulations detailed here were prepared by Method B; see text.

	Weight ratio SDS: alcohols	Total Emulsifier	C_16_OH	C_18_OH	SDS	1,5- C_5_OH_2_	Oil	H_2_O	D_2_O
		**Percentage % w/w**							
C3	1:9	4	1.70	1.90	**0.40 (50% D)**	—	20.00	97.00	3.00
C4	1:9	5	2.13	2.37	**0.50 (50% D)**	—	20.00	97.00	3.00
C5	1:9	7	2.98	3.32	**0.70 (50% D)**	—	20.00	97.00	3.00
C6	1:9	10	**4.25 (100% D)**	4.75	1.00	—	20.00	96.00	4.00
C7	1:9	10	4.25	**4.75 (100% D)**	1.00	—	20.00	96.00	4.00
C8	1:19	10	4.49	5.01	**0.50 (50% D)**	—	20.00	97.00	3.00
C9	1:9	10	4.25	4.75	**1.00 (100% D)**	—	20.00	96.00	4.00
C10	1:4	10	3.78	4.22	**2.00 (50% D)**	—	20.00	97.00	3.00
C11	1:9	10	4.25	4.75	1.00	**10.00 (100% D)**	20.00	96.00	4.00
C12	1:9	10	4.25	4.75	1.00	—	20.00	—	100
C13	1:9	10	4.25	4.75	1.00	10.00	20.00	—	100

### Method of preparation (A)

Creams were prepared using a Fundamix vibromixer. The oily and aqueous phases were heated separately to ~80 °C. SDS was dispersed in the aqueous phase at 80 °C and the dispersion mixed at a moderate speed for ~3 minutes. The oily phase was then transferred to the aqueous phase and the sample mixed at high speed with the temperature maintained at 80 °C. After ~3 minutes, the mixture was allowed to cool to room temperature with constant mixing.

### Method of preparation (B)

Creams were also prepared using an alternative method (which allowed for production of smaller volumes of sample), employing both a T18 digital Ultra-turrax disperser and Fundamix vibromixer. The oily and aqueous phases were heated separately to ~80 °C. SDS was dispersed in the aqueous phase at 80 °C before mixing at 13 000 rpm for ~3 minutes using the Ultra-turrax disperser. The oily phase was then transferred to the aqueous phase and the sample mixed at ~8 000 rpm (using the Ultra-turrax) with the temperature maintained at 80 °C. After ~3 minutes, the mixture was allowed to cool to room temperature, with constant mixing using the vibromixer.

### Microscopy

Creams were viewed under a microscope (Leitz Dialux 22 EB) fitted with a digital camera (Zeiss AxioCam HRc). A pin-tip amount of the sample was smeared onto a glass microscope slide and made as thin as possible by covering and pressing down with a cover slip. A 40× magnification lens was used to view the samples under normal light, and then under polarised light (using polarising filters) to detect birefringence.

### Rheology

Viscosity measurements were made at room temperature (25 °C) on an ARES rheometer (TA instruments) using parallel plate geometry (diameter 25 mm, gap 2 mm). Samples were transferred by spatula onto the centre of the plate, the geometry then lowered until it touched the sample and after the removal of any excess, the sample was allowed to rest for 3 minutes. Viscosity measurements were then made as a function of shear rate, over the range 0.01–100 (s^−1^) (logarithmic mode). All measurements were made in triplicate.

To aid comparison between different cream formulations, the linear portion of each viscosity flow curve (covering data obtained for shear rates in the range 0.01 to 1 s^−1^) was fitted according to the power-law model: $\,\eta =K{\dot{\gamma }}^{n-1}$ where $\eta$ is the cream viscosity (Pa.s), $\dot{\gamma }$ is the shear rate (s^−1^), *K* is the flow consistency index (Pa.s^n^) and $n$ is the flow behaviour index^47^.

### Small angle neutron scattering (SANS)

SANS measurements were variously performed on the LoQ and SANS2d beamlines at the ISIS pulsed neutron source (STFC Rutherford-Appleton Laboratory, UK), which employs neutrons separated by time-of-flight. LoQ uses neutrons of wavelength (λ) 2.2–10 Å, which are recorded at a 64 cm^2^ two-dimensional detector at a fixed distance of 4.1 m from the sample, giving a scattering vector (*Q* = (4π/λ) sin (θ)) in the range 0.008–0.22 Å^−1^. SANS2d uses neutrons of wavelengths 2–14 Å, which are recorded by a 96.5 cm^2^ two-dimensional detector at 8 m or 12 m from the sample, which gives a scattering vector in the range 0.0045 ≤ *Q* ≤ 0.4 Å^−1^.

Measured SANS data were processed using wavelength-dependent corrections to allow for the incident spectrum, detector efficiencies, and measured sample transmissions (as described in detail in Heenan *et al*.,^48^. SANS data were put on an absolute scale using the scattering from a standard sample (comprising a solid blend of protiated and perdeuterated polystyrene) in accordance with established procedures^49^.

In order to avoid possible shearing effects and mitigate issues with cell filling and emptying (as were encountered in pilot experiments), samples for SANS study were not loaded into the conventional Hellma cells but were instead loaded between two (21 mm diameter, 1.13 mm thick) silica windows, held at ~1 mm separation by a Teflon washer, and mounted in a bespoke sample holder (see Fig. S10, Supplementary information). Incident neutron beams of diameter 8 mm were used on LoQ and either 8 mm or 12 mm beams on SANS2d.

Initial SANS measurements were performed on LoQ. Here, 4% and 10% w/w emulsifier creams (compositions as given in Table 1) and their corresponding oil-free ternary systems were prepared using protiated organic components dispersed in 100% D_2_O. Subsequent SANS measurements were made on SANS2d using samples in which a single component was selectively deuterated in a 10% w/w emulsifier cream (namely either *d*_37_-octadecanol, *d*_33_-hexadecanol or *d*_25_-SDS, see Table 2) using a continuous phase of (96:4) or (97:3) v/v H_2_O:D_2_O (selected to contrast-match the average scattering length density of the protiated constituents in the creams). All measurements were performed at a temperature of 25 °C.

For all measured SANS profiles, checks were made to ensure that the pattern of scattering for each was the same for all incident neutron wavelengths and the samples were thereby confirmed to be free of multiple scattering. Checks were also performed to confirm that the SANS profiles were perfectly reproducible measurement-to-measurement and batch-to-batch, and showed no significant changes when prepared with Method A *vs*. Method B (see Fig. S1, and Table S1, Supplementary information).

SANS data for the *d*_25_-SDS cream were fitted to a core-shell oblate ellipsoid model^50^ using the SASVIEW package^51^. All other SANS data were fitted using Heenan’s FISH software^52^ using models which account for scattering from a thin surface and a 1-dimensional para-crystalline stack^53^ combined with a power law modelling^20^. A total of 11 parameters were model-fitted: the scale factors for the thin surface and para-crystalline stack, the thickness of the bilayers in the stack and the polydispersity on their thickness, the Lorentz bilayer, the mean number of bilayers *per* stack, the *d*-spacing of the bilayers in the stack and the polydispersity on this spacing, the coefficient and exponent for the power law model and the sample background. Uncertainties on the values of the fitted parameters are quoted as standard errors.

Given the complexity of the (bilayer/para-crystalline stack/power law) model used in fitting the SANS data, each dataset was fitted independently, but with the values of the fitted parameters checked so as to be consistent between the model-fits obtained for creams having the same composition but different H/D contrast.

Bragg peak positions for orders of reflection, *h*, were additionally obtained using the LAMP programme^54^ and the corresponding mean (lamellar) *d*-spacing calculated from a plot of *Q*/2π against *h*, and determination of the slope (1/*d*) by least-squares regression.

### Small angle and wide-angle X-ray scattering (SAXS and WAXS)

SAXS and WAXS measurements were made on a SAXS/WAXS Nano-inXider instrument (Xenocs, Sassenage, France) using a micro-focus sealed-tube Cu 30 W/30 µm X-ray source (Cu K-α, λ = 1.54 Å). The scattered X-rays (covering the Q range, 0.3 Å^−1^ to 4.1 Å^−1^) were detected using a Dectris Pilatus 3 hybrid pixel detector. Scattering from the samples was collected at room temperature.

The WAXS profiles were de-convoluted using LAMP^54^ and the positions of the component peaks obtained were used to calculate their corresponding *d*-spacings as *d *=2π/*Q*, where *d* is the in-plane lateral separation and *Q*, the scattering vector^14^.

## Supplementary information

Supplementary information

## Data Availability

The date obtained in the SANS experiments described here can be found at the DOIs: 10.5286/ISIS.E.RB1800007, 10.5286/ISIS.E.RB1710350, and 10.5286/ISIS.E.RB2000023.
