# Supplementary material for: Revealing the Hidden Details of Nanostructure in a Pharmaceutical Cream
Source: Sci Rep. 2020 Mar 5;10:4082. doi: 10.1038/s41598-020-61096-x (PMC7058068; doi:10.1038/s41598-020-61096-x)
Supplement: Supplementary file 1 — Supplementary information [file 41598_2020_61096_MOESM1_ESM.pdf]

Revealing the Hidden Details of Nanostructure in a Pharmaceutical Cream

Delaram Ahmadi[1], Najet Mahmoudi[2], Peixun Li[2], Kun Ma[2], James Doutch[2], Fabrizia Foglia[3], Richard K. Heenan[2], David Barlow[†1,4], M Jayne Lawrence[†,4]

1 Institute of Pharmaceutical Science, King's College London, Franklin Wilkins Building, 150 Stamford Street, London SE1 9NH
2 STFC ISIS Facility, Rutherford Appleton Laboratory, Chilton, Didcot, OX11 0QX
3 Department of Chemistry, Christopher Ingold Laboratories, University College London, Gordon Street, London WC1H 0AJ
4 Division of Pharmacy & Optometry, Stopford Building, University of Manchester, 99 Oxford Road, Manchester M13 9PG

Supplementary Information

(a)

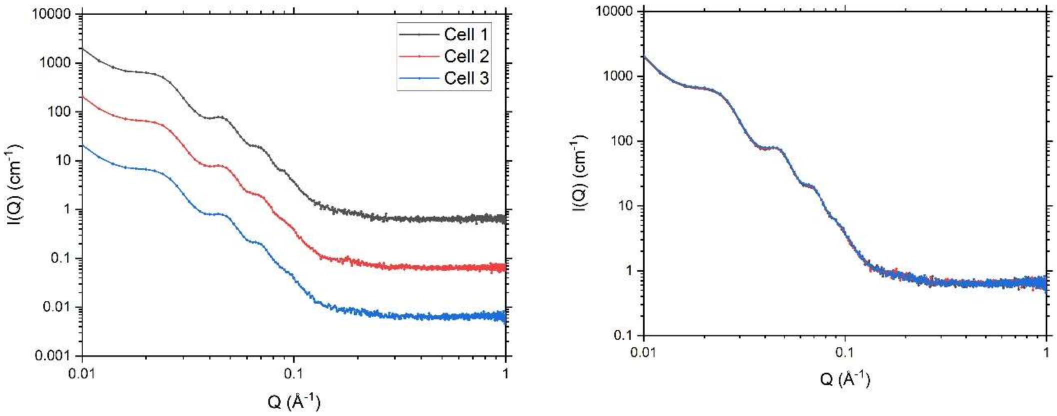

(b)

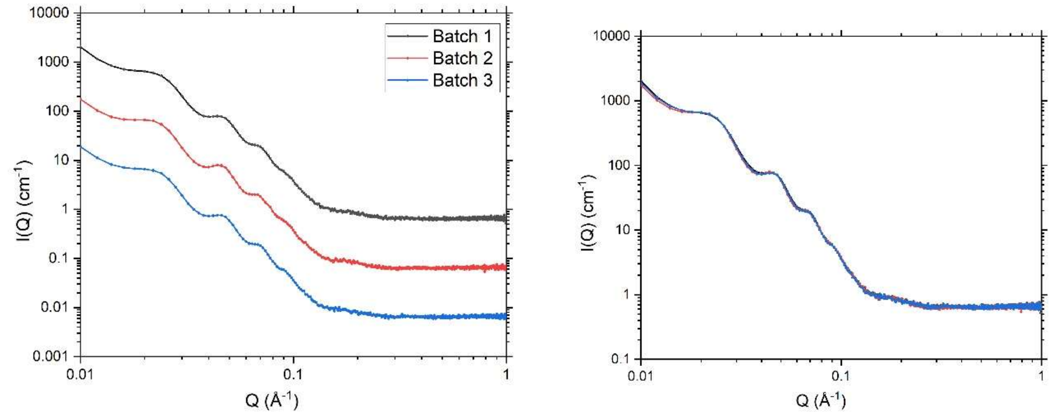

Fig. S1. (a) SANS profiles (overlaid at right, offset at left) obtained for three repeat sample cell preparations (black, red and blue) for batch 1 of a 10% emulsifier cream prepared using method B. (b) Averaged SANS profiles (overlaid at right, offset at left) of all cells (n=3) for three repeat batch preparations (black, red and blue) of 10% emulsifier creams prepared using method B. Method B: Aqueous and oily phases mixed using both a *T18* digital *Ultra-turrax*® disperser and Fundamix® vibromixer. Details of cream composition (C12) are as given in Methods, Table 2. Standard errors on the measured data are shown but are subsumed within the plotted symbols.

Table S1. Consistency indices (Pa.s) of 3 repeat preparations of 6-week old 10% emulsifier aqueous creams (C2; composition as given in Methods, Table 1) prepared using either method of preparation A or B. *Method A: Aqueous and oily phases mixed using Fundamix® vibromixer. Method B: Aqueous and oily phases mixed using both a *T18* digital *Ultra-turrax®* disperser and Fundamix® vibromixer.

	**Consistency index (K) (Pa.s)**		
**Method**	Batch 1	Batch 2	Batch 3
A	96±8	105±4	93±4
B	99±7	106±1	97±3

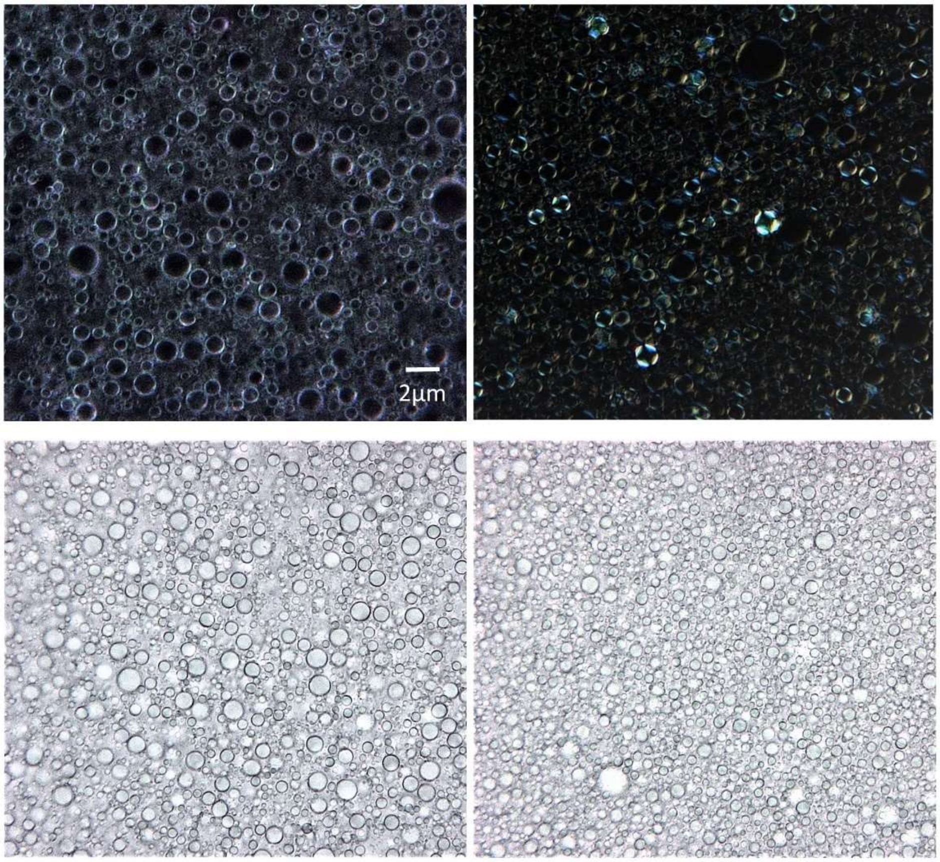

Fig. S2. Bright-field photomicrographs of freshly prepared 4% (C1) (lower left) and 10% (C2) (lower right) emulsifier creams, together with polarised light micrographs for the 4% and 10% creams (top left and right, respectively). The 4% cream shows a greater population of larger oil droplets and no evidence of Maltese crosses. Cream compositions are as given in Methods, Table 1.

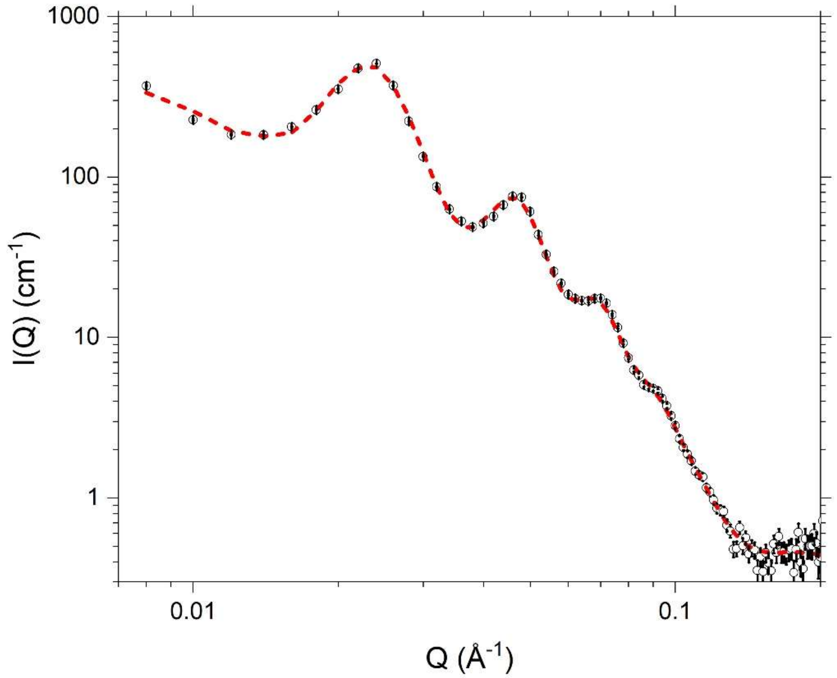

Fig. S3. SANS profile for a 10% emulsifier oil-free ternary system (black dotted circles) model-fitted (red) with a para-crystalline lamellar stack using FISH [52] (number of layers = 5, layer thickness (L) = 46 Å sig(L)/L = 0.17, *d*-spacing (D) =265 Å, sig(D)/D= 0.094). Standard errors on the measured data are shown but are subsumed within the plotted symbols.

(a)

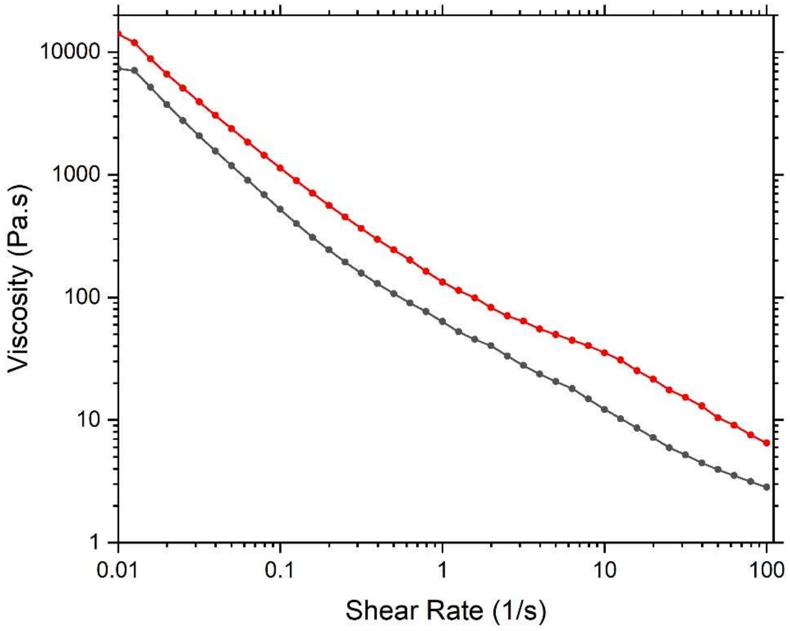

(b)

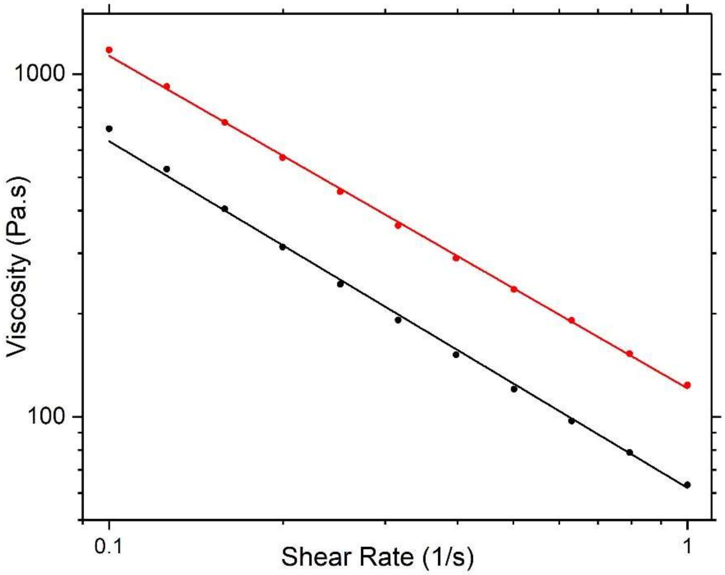

Fig. S4. (a) Full viscosity curve of a 4% (C1) (black) and 10% (C2) (red) emulsifier creams recorded for shear rates 0.01 – 100 s^{-1}**.** (b) power-law model applied to the viscosity curves of the 4% (red) and 10% (black) emulsifier creams over linear shear rates 0.1 – 1.0 s^{-1}, yielding consistency (K) indices of 61 Pa.s^n and 121 Pa.s^n, respectively. Cream compositions are as given in Methods, Table 1.

(a)

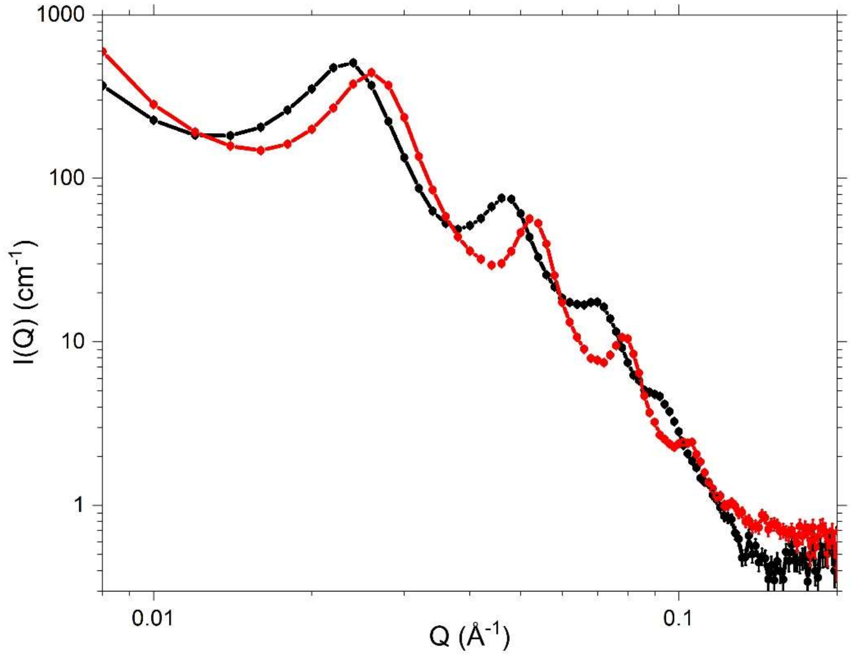

(b)

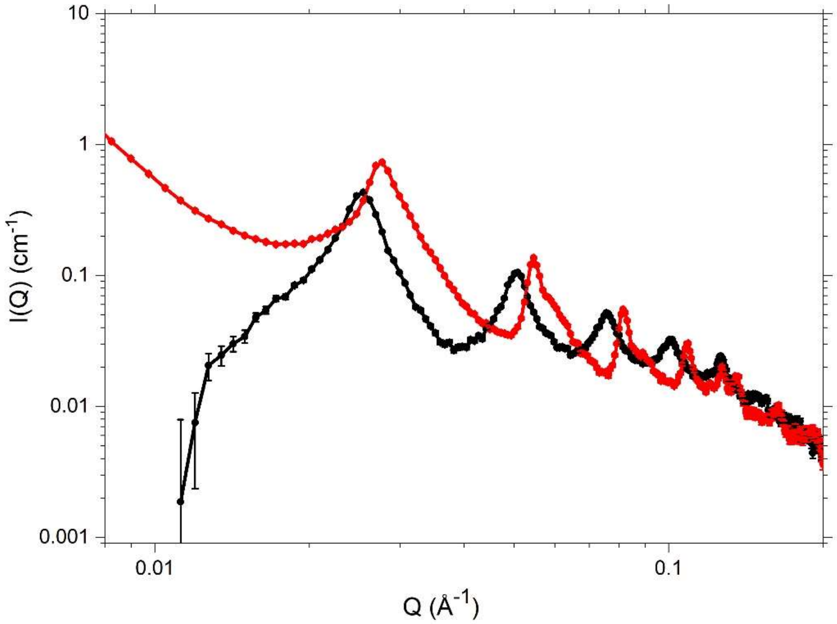

Fig. S5. (a) SANS and (b) SAXS profiles for a 10% emulsifier cream (C2) (red) and oil-free ternary system (black) at 25 °C with *d*-spacings obtained as 237 Å and 265 Å, respectively. Standard errors on the measured data are shown but are subsumed within the plotted symbols. Cream composition as given in Method, Table 1.

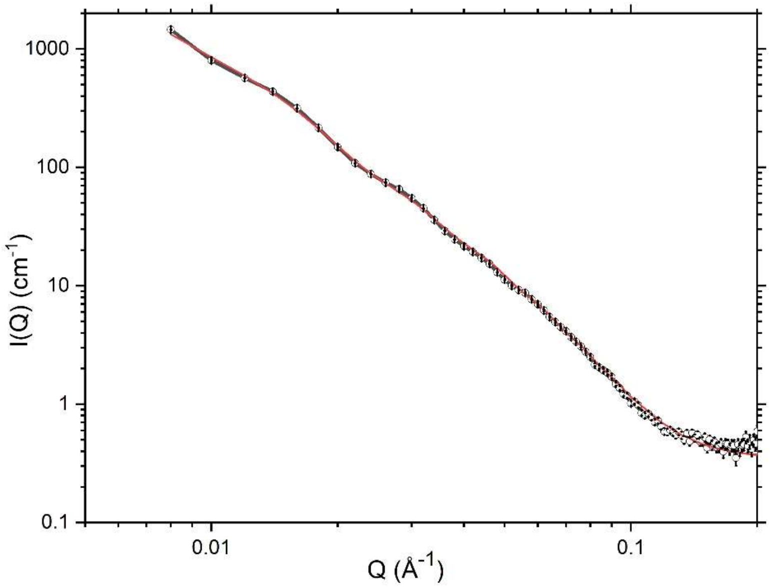

Fig. S6. SANS profile for a 4% (C1) emulsifier cream (black dotted circles) model-fitted (red) with a para-crystalline lamellar stack using FISH [52] (number of layers = 2, layer thickness (L) = 46 Å, sig(L)/L = 0.25, *d*-spacing (D) =286 Å, sig(D)/D= 0.29). Standard errors on the measured data are shown but are subsumed within the plotted symbols. Cream composition as given in Method, Table 1.

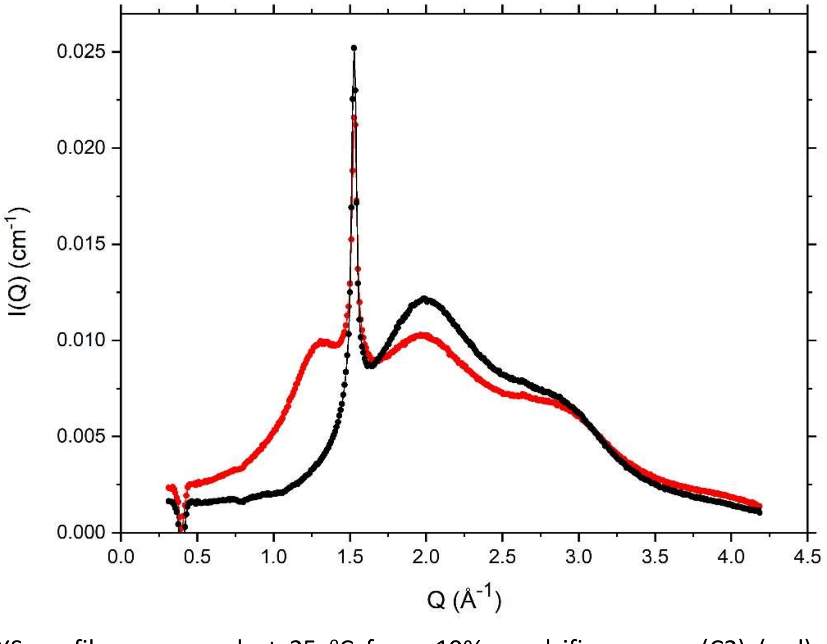

Fig. S7. WAXS profiles measured at 25 °C for a 10% emulsifier cream (C2) (red) and for the corresponding oil-free ternary system (black). Cream composition as given in Method, Table 1. Standard errors on the measured data are shown but are subsumed within the plotted symbols.

(a)

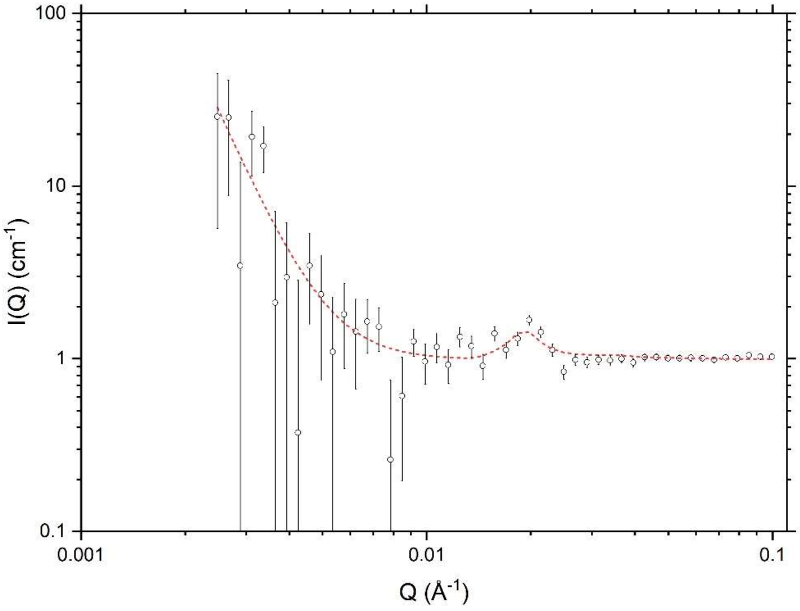

(b)

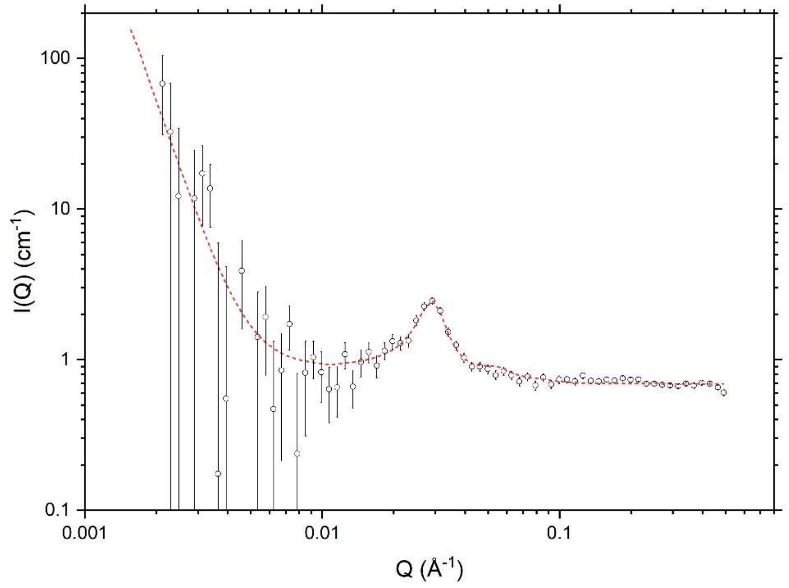

Fig. S8. SANS profiles recorded at 25 °C for creams prepared with 10% emulsifier and containing either 0.5% (C8) (a) or 2% (C10) (b) d_{25}-SDS such that the SDS:alkanols w/w ratio varies as 1:19 and 1:4, respectively. Cream compositions are as given in Method, Table 2.

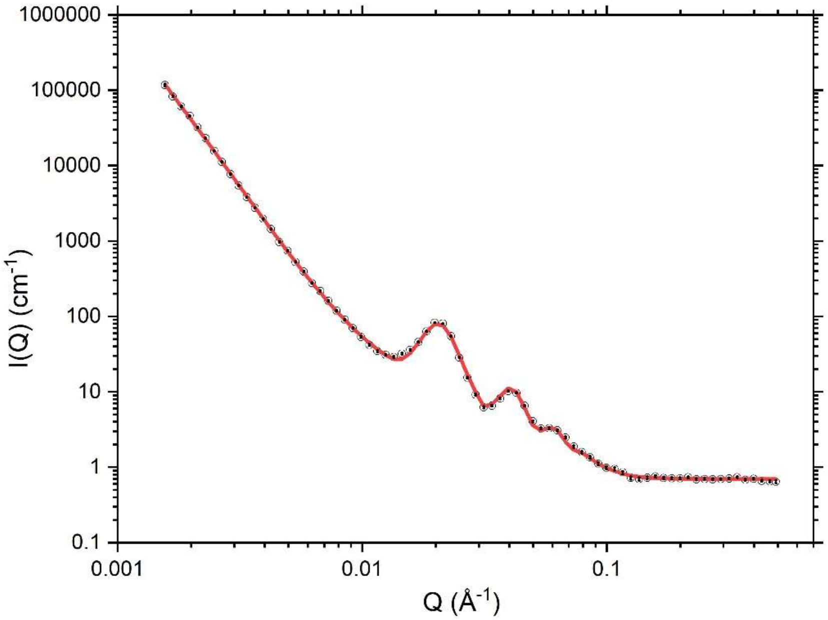

Fig. S9. SANS profile (black dotted circles) recorded at 25 ^{o}C for a 10% emulsifier cream containing 10% 1,5-pentanediol (C13), model-fitted (red) assuming lamellar stacks of bilayers of thickness 44.6 ± 1.6 Å, with a *d*-spacing of 306 ± 1 Å with a polydispersity of 0.09, combined with a power law modelling of the low-Q data with the exponent, -4.7 ± 0.1. Standard errors on the measured data are shown but are subsumed within the plotted symbols. Cream composition as given in Method, Table 2.

(1)

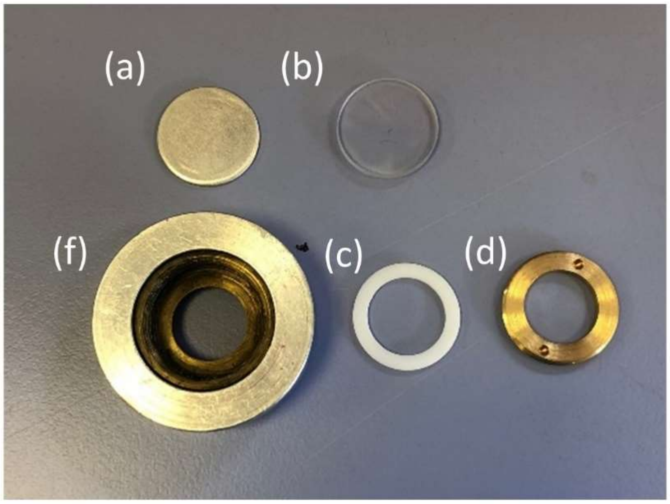

(2)

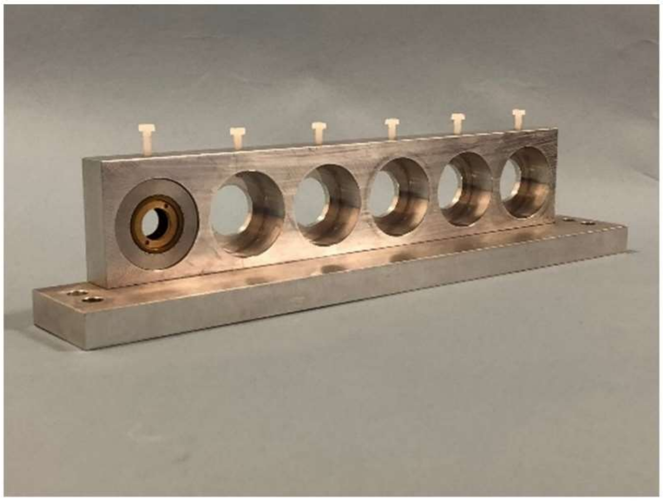

Fig. S10. The bespoke sample cells and sample rack used for SANS measurements (images 1 and 2, respectively). Samples were placed (a) between two silica windows (21 mm diameter, 1.13 mm thick) (b) separated by a Teflon washer (c) of ~1 mm width. (d) was then used to secure the sample assembly placed in (f). Image 2 shows an assembled cell mounted in the sample holder.
